# To Treat or not to Treat? The Fate of Patients with Intermittent Claudication Following Different Therapeutic Options

**DOI:** 10.31083/j.rcm2506229

**Published:** 2024-06-24

**Authors:** Elpiniki Tsolaki, Luca Traina, Caterina Savriè, Franco Guerzoni, Nicola Napoli, Roberto Manfredini, Maria Cristina Taddia, Fabio Manfredini, Nicola Lamberti

**Affiliations:** ^1^Unit of Vascular and Endovascular Surgery, University Hospital of Ferrara, 44124 Ferrara, Italy; ^2^Clinica Medica Unit, University Hospital of Ferrara, 44124 Ferrara, Italy; ^3^Health Statistics Unit, University Hospital of Ferrara, 44124 Ferrara, Italy; ^4^Department of Medical Sciences, University of Ferrara, 44124 Ferrara, Italy; ^5^Department of Neuroscience and Rehabilitation, University of Ferrara, 44121 Ferrara, Italy; ^6^Program of Vascular Rehabilitation and Exercise Medicine, University Hospital of Ferrara, 44124 Ferrara, Italy

**Keywords:** peripheral artery disease, exercise, intermittent claudication, vascular surgery, clinical outcomes, survival, hospitalizations

## Abstract

**Background:**

Peripheral artery disease (PAD) is recognized as a significant contributor to the public health burden in the cardiovascular field and has a significant rate of morbidity and mortality. In the intermediate stages, exercise therapy is recommended by the guidelines, although supervised programs are scarcely available. This single-center observational study aimed to evaluate the long-term outcomes of patients with PAD and claudication receiving optimal medical care and follow-up or revascularization procedures or structured home-based exercise.

**Methods:**

The records of 1590 PAD patients with claudication were assessed at the Vascular Surgery Unit between 2008 and 2017. Based on the findings of the recruitment visit, patients were assigned to one of the three following groups according to the available guidelines: Revascularization (Rev), structured exercise therapy (Ex), or control (Co). The exercise program was prescribed at the hospital and executed at home with two daily 10-minute interval walking sessions at a pain-free speed. The number and date of deaths, all-cause hospitalizations, and peripheral revascularizations for 5 years were collected from the Emilia-Romagna regional database.

**Results:**

At entry, 137 patients underwent revascularization; 1087 patients were included in the Ex group, and 366 were included in the Co group. At baseline, patients in the Rev group were significantly younger and had fewer comorbidities (*p* < 0.001). A propensity score matching analysis was performed, and three balanced subgroups of 119 patients were each created. The mortality rate was significantly (*p* < 0.001) greater in the Co (45%) group than in the Rev (11%) and Ex (11%) groups, as was the incidence of all-cause hospitalizations (Co: 95%; Rev 56%; Ex 60%; *p* < 0.001). There were no differences in peripheral revascularizations (Co: 19%; Rev: 17%; Ex 11%).

**Conclusions:**

In PAD patients with claudication, both revascularization procedures and structured home-based exercise sessions are associated with better long-term clinical outcomes than walking advice and follow-up only.

## 1. Introduction

Peripheral artery disease (PAD) in the lower extremities is a highly prevalent disease, particularly in individuals older than 50 years. Due to its rapid increase in incidence in lower-income countries and its impact on the functioning of older people in higher-income countries, PAD has currently become a global issue due to its social and economic consequences [1, 2, 3].

Underestimated for a long time, PAD has been recognized as a significant contributor to the public health burden in the cardiovascular field [3, 4, 5] and is considered responsible for 20% of all hospitalizations in the United States [6]. Indeed, PAD, as an expression of a generalized atherosclerotic process, is associated with an increased risk of cardio-cerebrovascular disease and related clinical outcomes and is 2–6 times higher than that of peers [2, 4, 7, 8]. In addition, in the intermediate stages of PAD, mobility, the ability to work, and health-related quality of life are influenced by intermittent claudication (IC), a common symptom represented by cramp-like pain that affects the leg muscles during walking, which disappears with rest [4]. Indeed, IC discourages spontaneous physical activity, accelerates functional decline [9, 10, 11, 12], and impacts the quality of life, the risk of falls, the rate of hospitalization, and related costs [6, 13].

In addition to necessary conservative management based on medical therapy, lifestyle modification, and risk factor reduction, revascularization interventions and rehabilitation therapy play significant roles in improving walking distance and limb salvage [2, 4, 14, 15]. However, as emphasized by consensus documents, it is essential to implement the correct procedure for the right patient at the right time, considering the objectives of reducing the risk of future cardiovascular events, improving walking performance, mobility, and quality of life, and ensuring limb preservation or pain reduction in the more severe PAD stages [16]. It is necessary to follow the most cost-effective strategy whenever possible and exploit the skills of an interdisciplinary team of professionals [4]. In particular, according to the principal guidelines, while the endovascular approach is generally recommended for patients requiring revascularization [4, 17, 18], exercise therapy and lifestyle modifications should represent the primary interventions for patients at Leriche–Fontaine stages I–II [4, 19], considering that at least 3-month walking training programs were effective in increasing walking ability and reducing the severity of claudication [19, 20].

Indeed, exercise therapy is recommended according to two types of structured programs [4, 19]. Supervised exercise programs, such as supervised exercise therapy (SET, Strength Grade I, evidence A) [4], are conducted in a hospital where intermittent treadmill walking exercise is administered up to moderate–severe pain intensity for approximately 30 minutes and repeated three times a week for at least 12 weeks [20]. These programs proved effective in promoting functional recovery without significant hemodynamic changes [21] and with lower inclusiveness or benefit for subgroups of patients, such as those with restricted mobility or low pain tolerance, including women [20]. They also revealed organizational problems such as limited availability of facilities delivering the programs and low adherence by patients who frequently visit the hospital [20, 22, 23]. To overcome some barriers to exercise and encourage patient participation, alternative programs have been proposed at home or in a community setting, with an instructor prescribing a walk-to-pain treatment designed to resemble the supervised hospital-based treatment [22, 23, 24]. Structured home-based programs (Strength Grade II, evidence A) were recommended without available SET programs as of 2017 [4]. These programs have been found to improve walking and spontaneous physical activity in the short term, although they are less effective than supervised programs [4, 20] and are affected by unequivocal results [24, 25, 26]. Approximately 20 years ago, a pain-free structured home-based program prescribed in the hospital and carried out at home, the test in–train out (TiTo) program, was designed and tested for patients with PAD [27, 28, 29]. The program is now offered to patients in active care, providing alternative care to vascular surgery in the presence of gait impairment and good health conditions. Based on this specific local setting, this single-center study aimed to evaluate the long-term outcomes of patients with PAD and IC receiving one of three possible therapeutic options: optimal medical care and follow-up, revascularization procedures, or structured home-based exercise.

## 2. Materials and Methods

In this observational study, we analyzed a prospectively collected dataset of patients assessed at the Unit of Vascular and Endovascular Surgery at the University Hospital of Ferrara from 2008 to 2017. The CE-AVEC Ethics Committee approved the study (number 277/2019). The Strengthening the Reporting of Observational studies in Epidemiology (STROBE) guidelines were followed for the preparation of this manuscript.

### 2.1 Subjects

Male and female patients aged >18 years and affected by PAD at Leriche–Fontaine stage II (both A and B substages) and intermittent claudication were evaluated for participation. Patients were not considered for the study if they were unable to ambulate independently, had advanced cancer, were absolutely contraindicated to exercise training (e.g., unstable angina, severe heart failure, major amputation, etc.) or revascularization procedures, or had other known clinical conditions that would limit survival within 1 year.

During the screening visit with the vascular surgeon, patients were assigned to one of the three following groups according to the available guidelines: Revascularization (Rev), structured exercise therapy (Ex), or control (Co). The choice of surgical, endovascular, or hybrid technique was based on lesion location and extent, following the European Society of Vascular Surgery (ESVS) guidelines [19].

### 2.2 Interventions

All patients underwent optimization of medical therapy and were advised to quit smoking.

### 2.3 Revascularization Group

Patients referred for peripheral revascularization underwent computed tomography (CT) scan angiography and/or diagnostic angiography and received the best possible treatment according to radiological findings. After treatment, all patients received antiplatelet therapy and underwent clinical examination and ultrasound follow-up at 6 and 12 months. The same surgical team performed surgical and endovascular interventions.

### 2.4 Exercise Group

Patients referred to the vascular rehabilitation program received the TiTo home-based pain-free exercise program [28, 29, 30]. This structured exercise program was prescribed during approximately monthly hospital visits and executed at home.

The program was composed of two daily 10-minute intermittent walking sessions (with a 1:1 walk: rest ratio) at a prescribed speed, converted into a walking cadence (steps/min), and maintained at home using a metronome. The training speed, which was slower than the individual’s walking speed at baseline, was increased weekly until the subject’s habitual velocity was reached. The exercise program lasted 6 ± 1 months and encompassed five hospital visits. More details on the exercise program are reported elsewhere [28, 29, 30].

### 2.5 Control Group

Patients who were not candidates for revascularization or unwilling to participate in the exercise program were advised to walk at least 30 minutes for 5 days per week while enduring claudication pain.

### 2.6 Outcomes

All participants underwent periodic (approximately one time per year) follow-up controls at the Unit of Vascular and Endovascular Surgery.

Clinical status, the severity of PAD, medical history, and the ankle–brachial index were collected from the patient’s medical record. An independent researcher blinded to the patient-group allocations created the dataset using the clinical information.

Long-term clinical outcomes were obtained from the Emilia-Romagna Regional Health Service Registry. A 5-year follow-up period was considered.

The five-year survival probability was the primary outcome. Secondary outcomes included PAD-related lower limb revascularizations (including both endovascular and surgical procedures) and all-cause hospitalizations.

Outcomes were considered after the date of enrollment by the vascular surgeon. In the case of death, in the absence of a precise outcome (e.g., peripheral revascularization), the data were censored for the date of death.

### 2.7 Statistical Analysis

The data distribution was verified using the Shapiro‒Wilk test. Differences in baseline characteristics for the two groups were evaluated using chi-square tests, Student’s *t*-tests, or Mann‒Whitney tests, as appropriate.

Kaplan–Meier estimates of the time distribution from enrollment to the date of death and a log-rank test for trend were used to compare the curves of the patient groups. Multivariate Cox proportional hazards regression analyses were employed to analyze the effect of several predictor variables on the primary outcome for each group. Owing to the limited number of events, multivariate hazard ratios (HRs) were calculated using a forward approach, with an entry limit of *p* < 0.05. A *p*-value < 0.05 was considered statistically significant.

All the statistical analyses were performed using MedCalc Statistical Software version 22.016 (MedCalc Software bvba, Ostend, Belgium).

## 3. Results

**Fig. 1.**
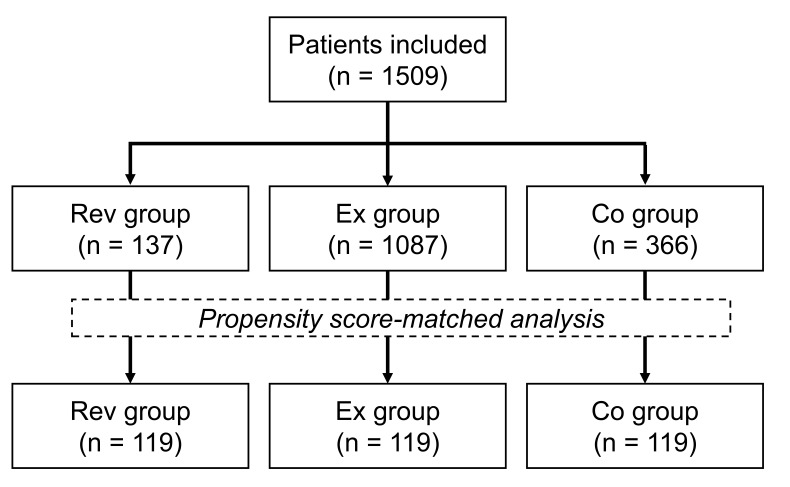
**Study flow diagram.** Rev, revascularization; Ex, structured exercise therapy; Co, control.

This analysis included 1590 patients who fulfilled the inclusion criteria. Of those, 137 underwent peripheral Rev, 1087 were included in the Ex group, and 366 patients who underwent reimaging comprised the control group. Fig. 1 reports a flow diagram of the study.

At baseline, patients in the Rev group were significantly younger (*p* < 0.001) and had a lower incidence of hypertension (*p* < 0.001), diabetes (*p* < 0.001), and chronic kidney disease (*p* < 0.001). The Charlson comorbidity index was also significantly lower in these patients (*p* < 0.001). These data are reported in Table 1.

**Table 1.** **Baseline comparison of the three groups under study**.

	Revascularization	Exercise	Control	*p*
	(n = 137)	(n = 1087)	(n = 366)	
Age, years	67 ± 8 †	71 ± 9	72 ± 10	<0.001
Male sex	103 (75%)	804 (74%)	267 (73%)	0.57
Risk factors				
Hypertension	103 (75%) †	957 (88%)	311 (85%)	<0.001
Hyperlipidemia	116 (85%)	902 (83%)	296 (81%)	0.42
Diabetes	58 (42%) †	533 (49%)	187 (51%)	<0.001
Chronic kidney disease	26 (19%) †	272 (25%)	91 (25%)	<0.001
Smoking	113 (82%)	924 (85%)	282 (84%)	0.18
Comorbidities				
Ischemic heart disease	76 (55%)	554 (51%)	194 (53%)	0.38
COPD	13 (9%)	120 (11%)	48 (13%)	0.31
Malignancies	22 (16%) †	217 (20%)	77 (21%)	0.031
Stroke/TIA	27 (20%)	196 (18%)	70 (19%)	0.67
Osteoarticular disorders	58 (42%)	402 (37%)	146 (40%)	0.24
Charlson index	4 ± 2 †	5 ± 3	6 ± 3	<0.001
ABI worse limb*	0.57 ± 0.25	0.58 ± 0.21	0.62 ± 0.24	0.08
ABI better limb*	0.77 ± 0.23	0.80 ± 0.21	0.78 ± 0.23	0.32

* ABI value was obtained only from the Co group in a population sample. † Different from the exercise and control groups. COPD, chronic obstructive pulmonary disease; TIA, transient ischemic attack; ABI, ankle-brachial index; Co, control.

### 3.1 Intervention Outcomes

All patients in the Rev group underwent the scheduled intervention without any perioperative mortality or amputation.

All patients in the Ex group started the rehabilitation program, with 91% completing it. A total of 98 patients interrupted the exercise program, mainly due to intercurrent disease or familial issues; two patients withdrew due to a lack of interest. Very good adherence to the home-based exercise was recorded, with a mean completion rate for the walking sessions of 85%. In the Co group, 72% of patients completed the scheduled vascular follow-up, reporting increased physical activity, although this was not measured.

To ensure balance among the three groups, a propensity score matching analysis was performed, resulting in three groups, each composed of 119 patients matched for baseline data. However, the Rev group was slightly younger, although the difference was not significant. The data are reported in Table 2.

**Table 2.** **Propensity-matched adjusted baseline comparisons among the three groups under study**.

		Revascularization	Exercise	Control	*p*
		(n = 119)	(n = 119)	(n = 119)	
Age, years		70 ± 9	71 ± 9	71 ± 10	0.16
Male sex		80 (67%)	90 (76%)	91 (76%)	0.37
Risk factors					
Hypertension		94 (79%)	95 (80%)	96 (81%)	0.39
Hyperlipidemia		80 (67%)	76 (64%)	74 (62%)	0.57
Diabetes		42 (35%)	47 (39%)	40 (34%)	0.18
Chronic kidney disease		22 (18%)	25 (21%)	26 (22%)	0.59
	Stages I–II	14 (12%)	12 (10%)	15 (13%)	
	Stages III–IV	8 (7%)	11 (9%)	10 (8%)	
	Stage V on dialysis	0 (0%)	2 (2%)	1 (1%)	
Smoking		99 (83%)	98 (82%)	100 (84%)	0.87
Comorbidities					
Ischemic heart disease		55 (46%)	59 (50%)	58 (49%)	0.21
COPD		13 (11%)	14 (12%)	16 (13%)	0.59
Malignancies		18 (15%)	19 (16%)	21 (18%)	0.87
Stroke/TIA		26 (22%)	18 (15%)	24 (20%)	0.19
Osteoarticular disorders		37 (31%)	41 (35%)	42 (35%)	0.39
Charlson index		4 ± 2	4 ± 2	4 ± 2	0.12
Lesions’ district					0.31
	Aortoiliac	30 (25%)	32 (27%)	29 (24%)	
	Femoropopliteal	96 (81%)	105 (88%)	100 (84%)	
	Subpopliteal	63 (53%)	58 (49%)	52 (44%)	
ABI worse limb*		0.59 ± 0.21	0.61 ± 0.19	0.60 ± 0.18	0.36
ABI better limb*		0.81 ± 0.16	0.81 ± 0.18	0.80 ± 0.22	0.42

* ABI value was obtained only from the Co group in a population sample. COPD, chronic obstructive pulmonary disease; TIA, transient ischemic attack; ABI, ankle-brachial index.

### 3.2 Primary Outcome

**Fig. 2.**
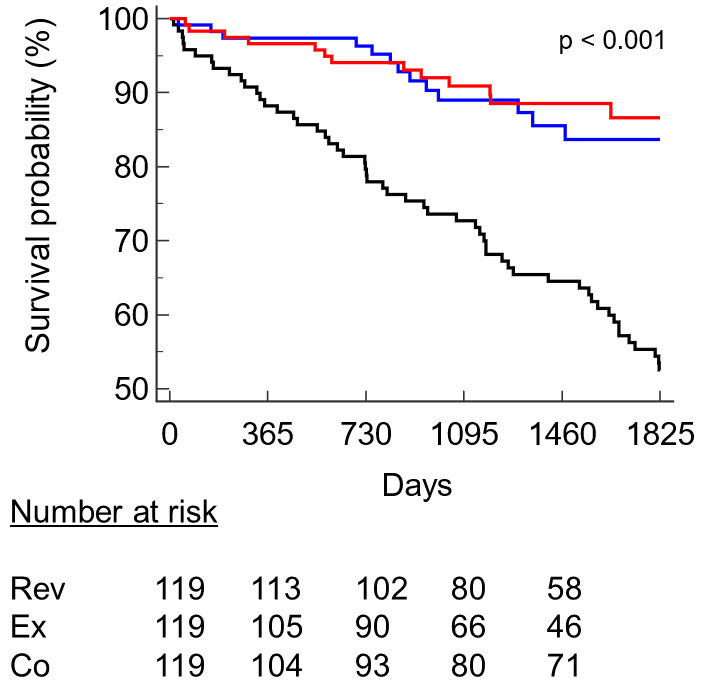
**Kaplan–Meier survival probability curve in the three groups.** Legend: red, revascularization; blue, exercise; black, control. Rev, revascularization; Ex, structured exercise therapy; Co, control.

At 5 years, mortality was significantly greater in the Co group, with a total of 53 deaths (45%) compared to the other two groups, which both exhibited a total of 13 deaths (11%). Therefore, the Co group had a greater HR than both the Rev (3.87; 95% CI 2.28 to 6.55) and Ex (3.39; 95% CI 1.96 to 5.84) groups. No differences were found between the revascularization and rehabilitation groups (Fig. 2).

### 3.3 Secondary Outcomes

**Fig. 3.**
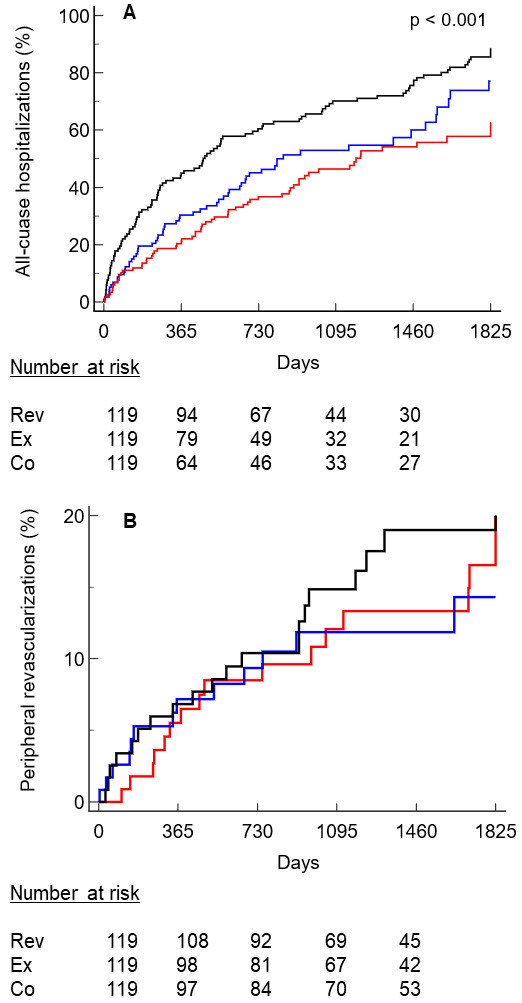
**The Kaplan–Meier curve of freedom from all-cause hospitalization (panel A) and peripheral revascularization (panel B) in the three groups. **Legend: red, revascularization; blue, exercise; black, control. Rev, revascularization; Ex, structured exercise therapy; Co, control.

All-cause hospital admissions at 5 years were greater in the Co group (n = 111; 95%) than in the Rev (n = 61; 56%) and the Ex groups (n = 71; 60%) (*p <* 0.001). The reasons for rehospitalization were principally due to cardiovascular issues (Co group: 46%; Rev group 39%; Ex group: 38%; *p =* 0.12) or internal medicine issues (Co group: 22%; Rev group: 19%; Ex group: 21%). The Co group presented a greater HR than both the Rev (2.18; 95% CI 1.61 to 1.94) and Ex groups (1.42; 95% CI 1.03 to 1.96). No significant differences were observed between the Rev and Ex groups (Fig. 3A).

Lower limb revascularization at 5 years was necessary for 23 (19%) patients in the Co group, 20 (17%) in the Rev group, and 3 (11%) in the Ex group, without any between-group differences (Fig. 3B). However, patients in the Co group who underwent revascularization had almost significantly lower mortality than their non-treated peers (HR: 0.54; 95% CI 0.29 to 1.01; *p = *0.053) (**Supplementary Fig. 1**).

### 3.4 Cox Proportional Hazard

Multivariate Cox regression analyses were used to explore the predictors of survival and identified age (HR 1.03; 1.01–1.04), chronic kidney disease (HR 1.81; 1.24–4.11), history of myocardial infarction (HR 1.52; 1.23–1.99) and belonging to the Co group (HR 2.88; 1.74–5.41) as independent risk factors.

## 4. Discussion

This study validates the increased morbidity in patients with PAD and highlights the positive impact of interventions that lead to functional recovery.

The primary finding of this study is the superiority of both treatments (surgical/endovascular treatment and exercise) over usual care (walking advice and follow-up) in terms of mortality and hospital admissions. The survival rates observed in both the rehabilitation and revascularization groups were similar but significantly greater than those in the control group.

Revascularization rapidly improves a fundamental component of functional status, the ability to walk, which enables individuals to perform normal daily activities. This active lifestyle may regulate cardiovascular risk factors such as diabetes mellitus, hypertension, and hypercholesterolemia, contributing significantly to the secondary prevention of cardiovascular diseases [31].

At 5 years, approximately 11% of the patients in the Rev group and 45% of those in the Co group died. By not comparing ever-identical populations, other studies have reported a 2-year cumulative mortality rate of 11.3% (7.8–24%) after revascularization in patients at different stages [32] or 54% of the patients (mean age 77 years at various clinical stages) who died from all-causes at a mean follow-up of 2.72 years after femoropopliteal revascularization [33]. Additionally, the present study showed that for 20% of the patients in the revascularization group, a second intervention was required within 5 years after the first procedure and predominantly after the third year. Such reintervention was successful for 75% of the patients with the same limb and 25% with the contralateral limb. High rates of recurrent rehospitalization and repeated revascularization procedures are expected within 2 years following the first peripheral revascularization [33], also considering the residual severe difficulty or inability to walk for two blocks, as reported in one out of five patients with claudication after endovascular treatment [34]. On the other hand, when successful, the revascularization procedure in PAD patients, in addition to improving functional status, significantly reduces the occurrence of future major cardiovascular events by approximately four times [35]. Moreover, the revascularization procedures, enhancing patients’ mobility [36], improved their prognosis and survival probability at the intermediate PAD stages [37, 38], although not in patients with critical limb ischemia [39].

A further important observation is that the Ex group had better outcomes than the usual care group and was superimposed on the Rev group. These results may be related to the positive effects of rehabilitation combined with modifying risk factors and lifestyle. In the Ex group, monthly check-ups promoted adherence to the treatment protocol, sustained motivation for a lifestyle change, and education on staying active after discharge. The Ex group was also characterized by fewer vascular procedures than the revascularization group, probably due to the systemic effect of the structured exercise. In particular, the TiTo program, administered to the subjects in the present study, was found to be effective at determining hemodynamic peripheral adaptations in the arteries of both the lower and upper limbs [40, 41, 42, 43, 44], resulting in a low revascularization rate 3 years after discharge from the program [45].

In addition, frail individuals, such as those on hemodialysis [46] with osteoarticular limitations and females [47, 48], were also included in this program, as they exhibited a high rate of adhesion and consistent functional recovery.

The results observed in this study may be related to different features of the program: (i) it is generally free for older populations and most patients; (ii) it requires only 4–6 hospital visits in a 6-month treatment period with limited transportation needs; (iii) it takes place in a home setting without weather limitations; especially, (iv) it is performed in the absence of pain due to low-intensity exercise and the particular design of the training sessions. These factors, together with the empowerment of patients through close monitoring and support in providing information on health issues and adherence to exercise therapy, may also explain the lower rate of hospitalization observed in these patients than in those in the usual care group [27, 28, 29].

According to the Leriche–Fontaine classification, the challenge in patients with Stage II PAD lies in ascertaining the actual benefits of each intervention. These patients pose numerous complex treatment issues, such as the degree of functional impairment and the extent of improvement necessary to achieve a perceived functional benefit; other factors to consider are represented by arterial anatomy and concomitant systemic diseases, as well as the local availability of a rehabilitation service. Additionally, patients’ motivation to adhere to exercise as a primary therapy plays an important role in therapeutic success. Finally, related incentives, restrictions, and treatment costs also impact patients’ therapeutic choices [49, 50].

This study highlights the crucial roles of both vascular medicine specialists (including surgeons and angiologists) and integrated facilities in receiving patients with broad recruitment criteria and initiating noninvasive recovery via a mobility program [51, 52]. This study may have several limitations due to its retrospective nature and treatment bias. The allocation of patients to specific treatment groups was not random. Instead, choosing a particular therapeutic strategy was based on various factors not analyzed in this report, including radiological findings, the patient’s health condition, pharmacologic therapy, and unresponsiveness to exercise. Furthermore, the patient’s preferences were also considered, especially concerning working issues. Physical function data or fitness habits of patients, as well as data about frailty status [53], left-ventricular ejection fraction, or B-type natriuretic peptide levels, were not collected at entry; instead, the analysis focused on clinical outcomes. The cause of death was not retrievable from the regional dataset. Finally, this study included a small cohort of patients without significant ethnic differences and a unique rehabilitation model that induced positive hemodynamic adaptations, which are generally unreported [21]. Therefore, these data cannot be generalized to all cohorts under study or rehabilitation programs.

## 5. Conclusions

This study showed that in patients with claudication, both revascularization procedures and structured home-based exercise sessions are associated with better long-term clinical outcomes than walking advice and follow-up only. Structured home-based exercise therapy represents a valid therapeutic option by contributing to the correction of risk factors, changes in lifestyle, and patient empowerment. The synergy between invasive and noninvasive interventions can benefit surgeons, patients, and families. Further studies are necessary to determine the causes of nonresponse to individual treatments to facilitate the first choice but also to investigate the role of exercise in patients at more severe stages of PAD or in combination with revascularization procedures.

## Data Availability

The datasets used and/or analyzed during the current study are available from the corresponding author on reasonable request.

## Supplementary Material

Supplementary material associated with this article can be found, in the online version, at https://doi.org/10.31083/j.rcm2506229.
