# Supplementary material for: To Treat or not to Treat? The Fate of Patients with Intermittent Claudication Following Different Therapeutic Options
Source: Rev Cardiovasc Med. 2024 Jun 24;25(6):229. doi: 10.31083/j.rcm2506229 (PMC11270107; doi:10.31083/j.rcm2506229)

Supplementary Fig. 1. Kaplan-Meier curve of survival probability comparing patients of Control group that underwent (dotted line) or not (continuous line) peripheral revascularization.


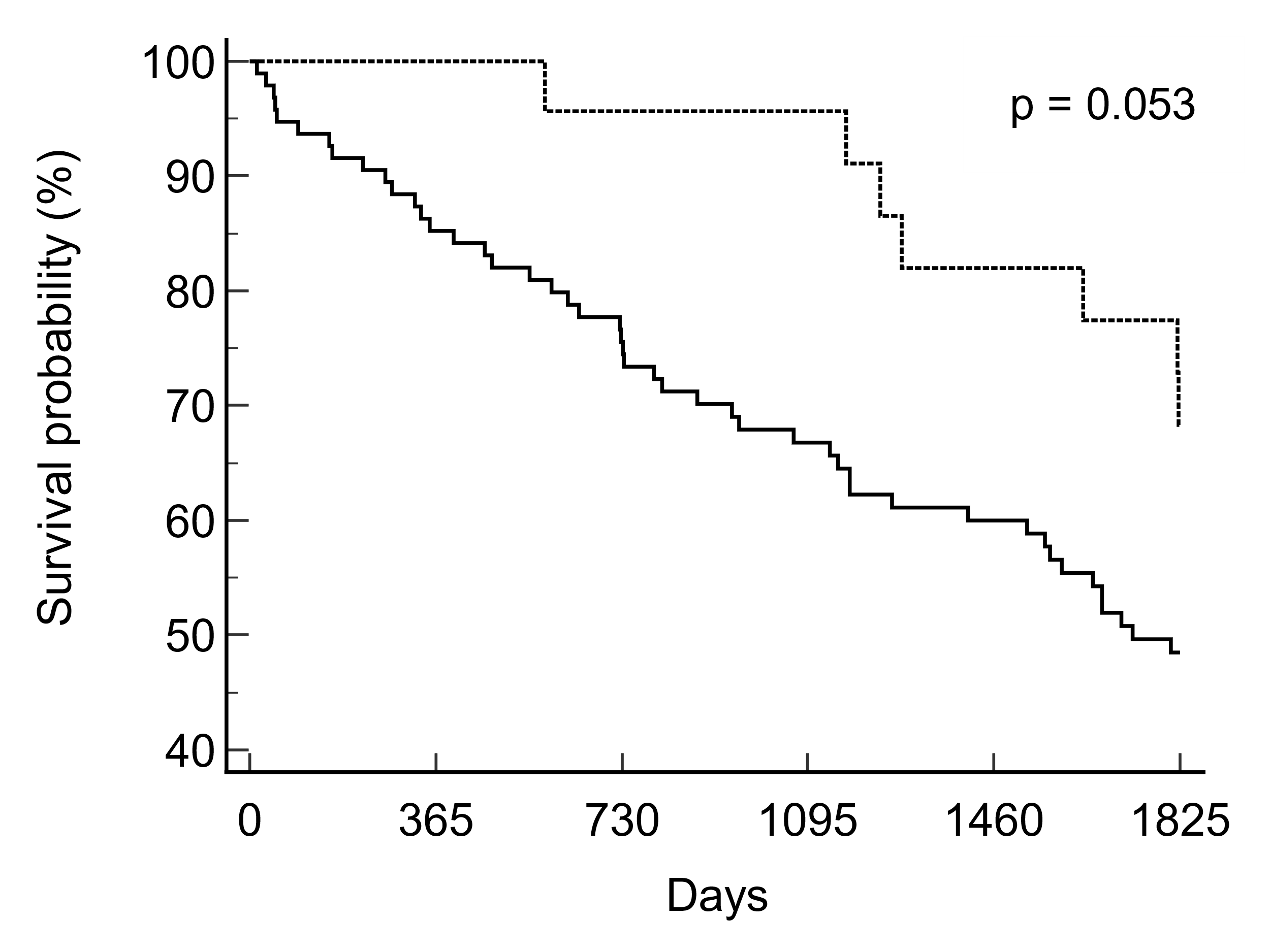

Supplement: Supplementary file 1 [file 2153-8174-25-6-229-s1.docx]
